# Electrospun nanofibers and their application as sensors for healthcare

**DOI:** 10.3389/fbioe.2025.1533367

**Published:** 2025-03-20

**Authors:** Yi-Sa Zhao, Jie Huang, Xingjian Yang, Weqiang Wang, Deng-Guang Yu, Hua He, Ping Liu, Kewei Du

**Affiliations:** ^1^ School of Health Science and Engineering, University of Shanghai for Science and Technology, Shanghai, China; ^2^ The Third Affiliated Hospital, Naval Medical University, Shanghai, China; ^3^ School of Materials and Chemistry, University of Shanghai for Science and Technology, Shanghai, China; ^4^ The Base of Achievement Transformation, Shidong Hospital Affiliated to University of Shanghai for Science and Technology, Shanghai, China; ^5^ Department of Orthopedics, Shidong Hospital Affiliated to University of Shanghai for Science and Technology, Shanghai, China

**Keywords:** electrospinning, nanofibers, sensor, influence parameter, healthcare

## Abstract

Electrospinning is a type of electrohydrodynamics that utilizes high-voltage electrostatic force to stretch a polymer solution into nanofibers under the influence of an electric field, with most of the fibers falling onto a collector. This technology is favored by researchers across various fields due to its simple and inexpensive device for producing nanofibers in a straightforward manner. Nanofibers prepared through electrospinning have a high specific surface area and high porosity. Electrospinning technology shows extensive potential, especially within biomedical sensors. This article provides a systematic overview of the factors influencing electrospinning, the parameters of the electrospinning process, the types of electrospun nanofibers, and the applications of electrospinning technology in the field of sensors, including wearable sensors, pressure sensors, and glucose sensors. The paper summarizes the research progress in this field and points out the direction of development for electrospinning technology, as well as the future challenges.

## 1 Introduction

Electrospinning, derived from “electrostatic spinning” (Qian et al., 2023; Javadi et al., 2024), produces nanofibers—though they often have diameters exceeding 100 nm (Yu et al., 2025; Dong et al., 2024). Its origins can be traced back to the first detailed description of a fiber preparation device using a high-voltage electrostatic field, which marked the beginning of electrospinning (Ding et al., 2025). From a scientific perspective, electrospinning is a special case of electrostatic atomization or electrospraying, a concept dating back to 1745. The key difference lies in the working media: electrostatic atomization uses low-viscosity Newtonian fluids, while electrospinning employs higher-viscosity non-Newtonian fluids (Ji et al., 2023; Mao et al., 2024; Chen S. et al., 2025). This distinction provides a theoretical basis for electrospinning systems. The process involves multiple fields, including electrostatics, electrohydrodynamics, rheology, and aerodynamics. From the 1930s to the 1980s, the development of electrospinning was slow, with most research focused on devices and patents that garnered little academic attention. However, after the 1990s, Reneker’s group at the University of Akron conducted extensive research on electrospinning and its applications (Sun et al., 2019). With advancements in nanotechnology, electrospinning has rapidly developed and attracted global interest. Electrospinning development can be divided into four overlapping stages. The first stage examines polymer spinnability, fiber diameter and performance, and process parameter optimization (Bai et al., 2022; Qosim et al., 2024). The second stage explores the composition and structural fine-tuning of electrospun nanofibers (Wang M. et al., 2025; Deng et al., 2025; Chen B. et al., 2025). The third stage focuses on applications in energy, environment, and biomedicine. The fourth stage investigates the batch manufacturing of electrospun fibers (Wang et al., 2024a; Wang et al., 2024b; Ding et al., 2025). Over the past decade, electrospinning has become a significant activity in materials science and technology. It offers advantages such as simple manufacturing, low cost, diverse spinnable substances, and controllable processes (Songfeng et al., 2023). Electrospinning can produce a wide variety of nanofibers, including organic, organic/inorganic composite, and inorganic materials (Lee et al., 2018). The fiber morphology depends on process parameters, material properties, and environmental conditions (Uhljar and Ambrus, 2023; Thompson, 2006). Electrospun polymer nanofibers (EPNFs) have high aspect ratios and excellent processability, utilizing various polymers and solvents (Al-Abduljabbar and Farooq, 2022). Electrospun nanofiber membranes are particularly valuable for biomedical applications due to their high specific surface area and tunable composition (Yan et al., 2022). This makes electrospinning a key technique for tissue engineering and drug delivery (Patel and Gundloori, 2023; Yu, 2025), as it can mimic the extracellular matrix and provide a cell-friendly environment (Elveren et al., 2023).

## 2 Electrospinning influencing factors

**FIGURE 1:**
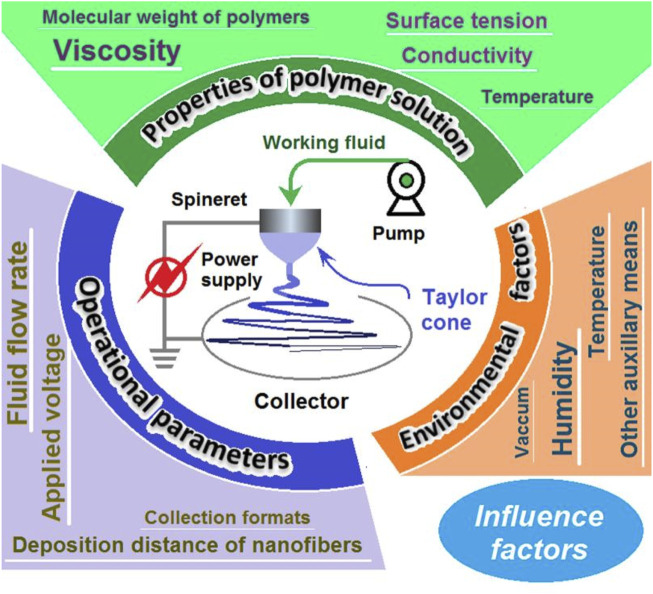
Main parameters affecting electrospinning and electrospun fibers.

Factors affecting electrospinning include the applied voltage, the distance from the nozzle to the collector, the rate of polymer injection, and the polymer concentration in the solution (Dolgin et al., 2023; Ziyadi et al., 2021), a diagram about the main parameters is shown in Figure 1. These parameters directly influence the formation and characteristics of the nanofibers produced (Abdulhussain, R., et al., 2023). Other factors include environmental conditions such as humidity, temperature, and syringe pump speed, which can significantly alter the electrospinning process and the final properties of the nanofibers (Ziyadi, H et al., 2021). In addition, the solution’s conductivity, viscosity, volatility, surface tension, and the strength of the electric field between the needle and the collector all play pivotal roles in the electrospinning process. These factors profoundly impact the size, morphology, and homogeneity of the nanofibers. For instance, the concentration and viscosity of the polymer solution affect the ease of electrospinning and the resulting fiber diameter (Dolgin et al., 2023; Xu et al., 2015). The mechanical and physical properties of nanofiber scaffolds, such as porosity, fiber diameter, crystallinity, mechanical strength, and topography, also significantly impact cell proliferation, migration, and differentiation (Xing et al., 2023). These properties are essential for the successful application of electrospun nanofibers in fields like tissue engineering and drug delivery, where the interaction between cells and the scaffold material is critical.

### 2.1 Polymer solution properties

#### 2.1.1 Molecular weight of polymers

Polymers and polymer-based composites are among the most important factors for develop new healthcare materials (Zhang et al., 2023a; Riaz et al., 2024; Gupta et al., 2023). The relative molecular weight of significantly influences electrospinning. Polymers with low relative molecular weights have weakly interacting molecular chains, leading to fragility and a tendency to break. This can cause defects such as feathering and adhesion during electrospinning, negatively impacting fiber quality. Conversely, high relative molecular weights result in extended chains that form spatial structures, like laminar patterns, which can hinder solution fluidity and lead to uneven stretching, weak tensile properties, and irregular fiber diameters. Therefore, an appropriate relative molecular weight is crucial for optimal electrospinning performance. Within this optimal range, molecular chain interactions are balanced, avoiding issues like leakage, adhesion, and non-uniform fiber diameters while maintaining solution fluidity to produce high-quality nanofibers. Ultra-high molecular weight polyethylene (UHMWPE) is a notable example. Different grades of UHMWPE have been used to study their effects on electrospun nanofibers, with the molecular weight as a key factor. Research has observed differences in the concentrations required fore optimal fiber formation between UM2.21 and UM4.43 (Yu et al., 2016). Despite these differences, the fiber diameter increases with viscosity in a nearly identical manner for both grades (Nayak et al., 2022), suggesting that while molecular weight is important, viscosity also significantly affects electrospinning and nanofiber characteristics.Molecular simulation can be a useful tool for predicting molecular weight and promoting the targeted applications of polymers (Chen et al., 2024a; Pan et al., 2024).

#### 2.1.2 Viscosity

Most of the current research on electrospinning is focused on the process conditions that govern the technique (Zhang et al., 2015; Lv et al., 2025). The characteristics of electrospun fibers and their potential applications are significantly influenced by the nature of the polymer solution, which plays a decisive role in the electrospinning process (Korycka et al., 2018). Among the properties of the polymer solution, viscosity is a key parameter. These properties can be controlled by adjusting the concentration of the polymer solution, a parameter that determines whether nanofibers can be successfully spun (Huang et al., 2003). Viscosity directly affects fiber diameter and morphology and is considered the main parameter determining nanofiber diameter and the ease of successful electrospinning (Amariei et al., 2017). The viscosity of the polymer solution for electrospinning should be controlled within a suitable range. If the solution’s viscosity is outside this range, successful electrospinning may not be possible. Fibers produced at the edge of the critical value may have poor morphology, or continuous nanofibers may not be easily obtained. When the solution viscosity is too high above the critical value, it may prevent the polymer from being extruded through the needles, leading to spinning failure. Within the appropriate range, higher viscosity can obstruct fiber drafting and thinning, resulting in larger fiber diameters and a wider diameter distribution. Conversely, too low a viscosity may lead to the interruption of polymer filaments and the formation of polymer droplets during electrospraying. Within the appropriate viscosity range, lower viscosity results in finer nanofibers. When the solution viscosity is below the critical value, it may cause nanofiber breakage due to electrospinning discontinuity. To achieve optimal electrospinning results, it is essential to find the balance in solution viscosity. This balance ensures that the polymer solution can be extruded and spun into fibers without breaking or forming droplets, and that the resulting fibers have the desired diameter and uniformity (Ravishankar P. et al., 2019). By carefully controlling the viscosity, researchers can tailor the properties of electrospun nanofibers to meet specific application requirements.

It has now been studied on polymers such as polylactic-co-glycolic acid (PLGA), polyethylene oxide (PEO), polyvinyl alcohol (PVA), polymethyl methacrylate (PMMA), polystyrene (PS), and poly-L-lactic acid (PLLA). The relationship between the viscosity of these polymers and their suitability for electrospinning is shown in Table 1.

**TABLE 1:** Relationship between viscosity and electrostatically spun polymers.

Polymer solutions	Viscosity-related	References
Gelatine	The viscosity should be within the range of 300–700 mPa s; viscosities higher than this range will increase the diameter of the nanofibers	Niehues and Quadri (2017)
PEO/NaAlg	Lower viscosity produces smaller but more numerous beads, resulting in a larger total area	Böncü (2023)
PAN	A high dynamic viscosity results in more uniform nanofibers	Mpukuta et al. (2020)
PCL	Viscosity affects the diameter of PCL nanofibers, with higher viscosity producing smaller diameter nanofibers	Chen et al. (2021)

#### 2.1.3 Surface tension

Surface tension, which often reflects changes in surface free energy per unit area, plays a decisive role in electrospinning. It significantly affects the efficiency of nanofiber production, particularly in free-surface electrospinning processes (Jiang et al., 2019). Changing the surface tension significantly affects the distance between the jets in electrospinning: as surface tension increases, the distance between the jets also increases, resulting in fewer jets being ejected. Conversely, a decrease in surface tension leads to a shorter distance between the electrospun jets, allowing more jets to appear in the process. Voltage changes also affect the number of jets, though not as markedly as surface tension changes. As voltage increases or decreases, the jet stream distance correspondingly increases or decreases. Surfactants can be added to the spinning solution to alter its surface tension (Jia and Qin, 2012). In the electrospinning process, the polymer solution must be charged sufficiently to overcome the surface tension. As the polymer solution jet accelerates from the spinneret to the collector, it stretches. The surface tension of the solution may cause the jet to disintegrate into droplets, leading to a process known as electrospraying. When the nanofibers produced by electrospinning are collected through this process, it is also referred to as electrospraying. High surface tension in the solvent system results in poor fiber morphology, particularly in collagen fibers produced by electrospinning (Dong et al., 2011). The surface tension of polymer solutions also affects the diameter of nanofibers produced by bubble electrospinning (Liu et al., 2010). To produce ultrafine fibers using bubble electrospinning, smaller bubbles require less electrostatic force (He and Liu, 2012).

#### 2.1.4 Conductivity

The conductivity of electrospun polymer solutions is directly related to their electron conduction capacity (Blachowicz and Ehrmann, 2019). As the polymer solution’s conductivity increases with its electron capacity, it directly affects the shape and appearance of the electrospun filament (Sheoran et al., 2021). Thus, the polymer solution’s conductivity can significantly affect the morphology and size of the nanofibers (Kafiah et al., 2022). Increasing the solution’s conductivity leads to a decrease in fiber diameter, resulting in the production of nanofibers with smaller diameters (Pavelkova et al., 2017). Conductive nanofiber mats can be produced using two methods: from mixtures of electrospinning agents with electrically conductive polymers or nanoparticles, and from conductive mapping layers (Mousa et al., 2022). The conductivity of nanofibers can be enhanced by blending conductive polymers with other spinnable polymers. This conductivity of the polymer solution also influences the quality of the resulting nanofibers, with higher conductivity typically leading to improved nanofiber quality. Overall, the polymer solution’s conductivity plays a crucial role in determining the morphology, size, and conductivity of the electrospun nanofibers.

Mousavi et al. (2016) investigated the optimization of the loading of two polymers in electrospinning by optimizing the loading of both polymers based on a poly (methyl methacrylate)/polyacrylonitrile (PMMA/PAN) blend. They produced a new fiber polymer electrolyte membrane, demonstrating that the conductivity of ions is related to the PMMA content and increases with the increase in PMMA content until it reaches 50%. As the PMMA content increases further, there is a smooth decrease in ionic conductivity, which thereby affects the nanofiber diameter. The electrical conductivity of homogeneous polyamide 6,6 (PA66) nanofibers can be increased by adding organic salts during the electrospinning preparation. For instance, increasing the conductivity by adding benzyltrimethylammonium chloride (C10H16ClN, BTMAC) to the solution results in the production of homogeneous nanofibers. In contrast, homogeneous nanofibers can only be obtained under limited conditions without the addition of organic salts. This suggests that the electrospinning process can be improved by increasing the conductivity of the solution (Ryu and Kwak, 2013).

#### 2.1.5 Properties of solutions

The nature of the solution indeed plays a decisive role in the electrospinning process. Properties such as conductivity, volatility, surface tension, viscosity, and molecular weight all impact the electrospinning process, which in turn influences the morphology of the nanofibers formed (Al-Abduljabbar and Farooq, 2022; Canjie et al., 2023; Zhou et al., 2023). As conductivity increases, the diameter of electrospun nanofibers typically decreases. The properties of the solutions used in electrospinning can significantly affect the process and the resulting nanofibers. The concentration of the polymer solution and the temperature of the prepared solution influence the rheological properties of the solution, which in turn affect the electrospinning parameters and the quality of the resulting nanofibers (Gonçalves et al., 2022). Different solvent ratios also affect the properties of nanofiber structures, including their physicochemical, microstructural, and thermal properties. For instance, using N,N-dimethylformamide (DMF) as a fiber differentiation inducer reduces the diameter and increases the crystallinity of electrospun fibers (Shen et al., 2022). Controlling the solution temperature during electrospinning allows for the use of different solvents, enabling the use of less toxic solvents and expanding the range of spinnable solvents (Abbas et al., 2022). In addition, adding specific additives to the solvent, such as aloe vera to a polyvinyl alcohol solution for electrospinning, allows for the microencapsulation of these additives within the fibers. This process imparts nutritional properties to the fibers, resulting in nanofibers with health benefits (Gospodinova and Neznakomova, 2022). Chitosan (CS) is a biodegradable and biocompatible polymer with antimicrobial and antioxidant properties. The cationic structure of chitosan has a fixed charge and an inherent performance viscosity, which affect the hydrodynamic properties of electrospinning solutions, even at very low concentrations. The structure of CS can be chemically modulated by increasing the degree of acetylation and the content of amino acids; this modification is used to improve the spinnability of the solution.

#### 2.1.6 Temperature

The temperature of the polymer solution affects not only the viscosity of the solution but also the volatilization of the solvent during the electrospinning process (Li X. et al., 2021). When the temperature of the polymer solution increases, the viscosity can be greatly reduced, while the entanglement of the molecular chains in the solution remains almost unaffected. Therefore, adjusting the temperature of the spinning solution can regulate the appearance of the nanofibers produced by electrospinning. Moreover, the polymer solution temperature influences surface tension and solvent evaporation during electrospinning.

The electrospinning polymer solution significantly affects the electrospinning process. For instance, with the amorphous polymer N-isopropylacrylamide (a-pNiPAM), the solution fluctuates at a temperature associated with high concentration fluctuations (T1), followed by gelation at the gelation temperature (Tgel). Then, at the binodal temperature (Tb), phase separation occurs. Similarly, for poly (vinylidene fluoride)-hexafluoropropylene (PVDF-HFP), precise control of the solution temperature is required for successful electrospinning. Different solvents require different temperatures (Abbas et al., 2022). The temperature of the polyacrylonitrile and methyl acrylate copolymer solution significantly affects both the viscosity of the solution and the homogeneity of the resulting nanofibers. This work investigated the effect of production temperature on polyacrylonitrile and methyl acrylate copolymer during spinning. The observable phenomenon was an inverse relationship between the preparation temperature of the solution and the viscosity of the solution at room temperature, primarily affecting the integrity and homogeneity of the fibers formed. In addition, the temperature in the titanium dioxide nanofiber synthesis process affects the photoactivity and efficiency of the resulting nanofibers (Silva and Alves, 2022). Overall, the temperature of the polymer solution plays a crucial role in determining the properties and characteristics of the resulting nanofibers from the electrospinning process.

### 2.2 Process parameters

#### 2.2.1 Input voltage

The electrospinning voltage is a crucial parameter for spinning nanofibers (Li X. et al., 2021). Within an appropriate voltage range, the prepared nanofibers are continuous and have a smooth surface. As the voltage increases, the diameter of the nanofibers becomes smaller, while a decrease in voltage results in larger diameter nanofibers. When the electrospinning voltage is too low, the electric field force generated is relatively weak, making it difficult to overcome the surface tension of the spinning solution. This difficulty hinders the stretching and splitting of the solution, leading to the formation of larger-diameter nanofibers. However, as the voltage is gradually increased, the electric field force becomes strong enough to overcome the solution’s surface tension. This enables the polymer solution to stretch and split more easily, resulting in the formation of nanofibers with finer diameters. The nanofibers become finer as they approach the optimal voltage range. Beyond this range, if the electrospinning voltage exceeds the maximum suitable value, the electric field strength becomes too large. This leads to an increase in the jet volume of the spinning liquid and accelerates the jet speed, which is not conducive to the stretching and splitting of the jet. Consequently, the diameter of the fibers becomes larger, their homogeneity decreases, and beaded or bead-like nanofibers may form.

The voltage applied during the electrospinning process significantly affects fiber morphology, crystallinity, and conductivity. Lower voltage leads to fiber thickening. Applying different voltages at different times results in significant changes in both average conductivity and average resistance. As the voltage increases, the average resistance decreases while the average conductivity increases (Budiarto et al., 2023). At higher voltages, the tension within the spinning jet increases, leading to more uniform fiber diameters and improved fiber uniformity (Safriani et al., 2022). The influence of applied voltage on nanofiber morphology was examined using electrospun polyvinylidene difluoride (PVDF). It was found that the fiber diameter is influenced by the deformation of the spinning jet, which is uniformly distributed at low voltages. In contrast, at higher voltages, the fiber diameter becomes more uniformly distributed. This improved uniformity of the fiber diameter is due to the increase in surface tension, which leads to the contraction of the Taylor Cone (Nugraha et al., 2022). Using non-DC voltages, such as AC and AC with DC components, in electrospinning has yielded positive results. Both AC and AC with DC components have been proven effective in this process (Cselkó et al., 2023). Productivity and the area covered by the fibers are also affected by the voltage level. In experiments using the electrospinning hair method to prepare silica, increasing the voltage results in more continuous silica nanofibers and fewer beads. As the voltage applied to the electrospinning process increases, the average fiber straightness also increases (Ginting et al., 2022). Overall, voltage plays an irreplaceable role in determining the properties of electrospun fibers.

#### 2.2.2 Flow rate of the solution

The solution flow rate is a key parameter in the electrospinning process, significantly affecting the nanofiber diameters, morphology, and properties. Generally, when the electrospinning solution flow rate is low, nanofiber formation is slower. This allows the electric field force generated by the high-voltage electric field more time to stretch the solution, resulting in the production of nanofibers with finer diameters. Consequently, this leads to nanofibers with a more homogeneous morphology. However, a higher flow rate may result in beaded nanofibers, affecting the yield and quality of the fibers. The solution flow rate is an important parameter in the electrospinning process, directly affecting fiber formation, diameter, morphology, and properties. By precisely controlling the flow rate, you can optimize the production process to obtain high-performance fibers for specific applications. When the solution flow rate is high, it accelerates the rate of nanofiber formation, and the tensile strength effect of the electric field becomes more pronounced. Such a result leads to coarser fiber diameters and more diverse nanofiber morphologies. While a high flow rate of the polymer solution can increase the productivity of the nanofiber capacitive solution, it also reduces the final morphology’s homogeneity, ultimately affecting their properties.

Different studies have shown that changes in the flow rate of the polymer solution affect the morphology, diameter, and other properties of electrospun nanofibers. The application of zinc oxide nanofibers in the fabrication of dye-sensitized solar cells (DSSCs) at relatively low solution flow rates results in smaller droplet sizes and a more homogeneous morphology of the DSSCs. This leads to higher electrical efficiencies for DSSCs. In the production process of obtaining poly (vinyl acetate) (PVAc) microfibers, increasing the electrospinning flow rate within a solution flow rate range of 2–5 mL/h results in larger nanofiber diameters. However, if the flow rate is too fast, the short drying time before reaching the collector leads to the formation of coarser-diameter, bead-like nanofibers rather than finer-diameter ones. Additionally, it increases the hydrophobicity of the PVAc (Acik et al., 2019). Not only that, but a higher solution flow rate also affects the fiber thickness of polyvinylidene fluoride (PVDF). When the flow rate is faster, the electrospinning time is short, resulting in an electrospun felt that is not thick enough. This, in turn, affects the mechanical properties and electrical conductivity of the nanofibers. At higher flow rates, the formation of beads occurs, and the diameter of the fibers also increases (Budiarto et al., 2023). Furthermore, in the case of nanofibers produced from poly (vinylidene fluoride-hexafluoropropylene) (P(VDF-HFP)), the flow rate ranges from 0.1 to 0.9 mL/h. This range indicates that the diameter of the nanofibers produced is continuous. The highest nanofiber diameter is observed at a flow rate of 0.7 mL/h (Nawae et al., 2021). The prepared P(VDF-HFP) nanofibers can function stably as fiber strain sensors, unaffected by sweat or water.

Overall, the properties of the fibers, including their strength and electrical conductivity, are affected by the flow rate. This leads to structural differences within the nanofibers, thereby affecting their properties.

#### 2.2.3 Receiving distance of fibers

The distance between the injector nozzle and the collector influences solvent evaporation. A shorter distance may prevent full solvent evaporation, leading to thicker nanofiber diameters. When the distance is longer, the solvent can fully evaporate, allowing for the production of nanofibers with finer diameters (Chen Y. et al., 2024; Xue et al., 2020). Drying before reaching the collector needs to occur at the right distance; if the distance is too far or too close, you tend to get beads and blocks. In the process of electrospinning, the fiber receiving distance significantly affects the characteristics of the nanofibers. As the distance from the nozzle to the receiver increases, the force exerted on the fiber decreases, leading to larger fiber diameters and weaker hydrophobicity (Mu et al., 2021). Elsewhere, the distance of the nanofibers to the collector affects the direct distribution and alignment of the fibers, and furthermore affects the degree and diameter distribution. Liu et al. demonstrated that the optimal nanofiber collection class distance was between 15 cm and 20 cm by preparing PAN nanofibers (Liu et al., 2014). The collection distance of the fibers affects both the nanofiber size and tensile strength. Increasing the distance decreases the tensile strength and the size of the nanofibers, while decreasing the distance increases the tensile strength of the nanofibers (Al-Okaidy and Waisi, 2023). In drug delivery and tissue engineering applications, the distance from the fiber to the collector is also critical. Controlling this optimal distance is conducive to achieving the best results (Eslamian et al., 2019). Receiver distance is a key parameter in the electrospinning process, affecting the final physical properties, morphology, diameter, and fiber orientation of the fibers. By controlling the receiver distance, one can optimize the production process of electrospun nanofibers.

#### 2.2.4 Collectors

The type of collector and operating parameters have a significant impact on the structure and properties of electrospun nanofibers (Zhang et al., 2024b; Zhou, et al., 2024b). By choosing the right type of collector and optimizing its parameters, the microstructural and mechanical properties of nanofibers can be tailored to meet the needs of specific applications. These studies provide important technical support for the application of electrospun nanofibers in fields such as tissue engineering and regenerative medicine.Three-dimensional collector: Nanofibers with specific microstructures, such as pore size, pore volume, and connectivity, can be fabricated using a three-dimensional collector. By controlling the moving speed of the collector, the thickness and porosity of the fibers can be adjusted, which affects the crystallinity and mechanical properties of the fibers (Chen et al., 2020).Cylindrical collectors: Modified cylindrical collectors can improve fiber orientation, especially in biomedical applications that require longitudinal orientation of fibers. The length of the conducting and non-conducting segments has a significant effect on fiber diameter and orientation (Malik et al., 2020).Rotating collector: The speed of the rotating collector affects the external structure, crystallinity, and mechanical properties of the fibers. Higher rotational speed helps improve the mechanical properties and stability of the fibers (Bkkar et al., 2023).Water bath collector: Interconnected macroporous nanofibers can be generated directly using a water bath as a collector. The solvent ratio and water bath temperature have a significant effect on the pore structure of the fibers (Zhu L. et al., 2020).Collector parameters also affect the properties of the nanofibers:Voltage and rotational speed: Increasing the voltage or rotational speed can enhance the crystallinity and light absorption of the fibers, as well as affect the mechanical properties and shrinkage of the fibers (Bkkar et al., 2023). Collector geometry: Collectors with complex geometries may lead to inhomogeneous electric fields, affecting the uniform deposition of fibers. By adjusting the shape and material of the collector, the coverage and thickness uniformity of the fibers can be improved (Eom et al., 2020).

### 2.3 Dimensionless numbers and machine learning (ML) models

ML models have shown great promise in predicting the performance of nanofibers in a variety of applications, including adsorption capacity, diameter control, and mechanical properties (Subeshan et al., 2024). These models utilize complex datasets and advanced algorithms to optimize the properties of nanofibers and enhance their utility in areas such as environmental remediation and materials science.Machine learning models, such as Gaussian Process Regression (GPR) and Extra Learning Tree (ELT), have been developed for predicting the adsorption capacity of electrostatically spun nanofiber membranes for estrogens, achieving high accuracy (*R* ^2^ = 0.999) (Yasir et al., 2024). Key parameters affecting adsorption, including temperature, dose, initial concentration, contact time, and pH, were identified through Shapley analysis.Additionally, various machine learning algorithms, including Random Forest (RF) and Extra Tree Regression (ETR), have been used to predict the diameter of electrostatically spun nanofibers, with RF achieving an *R* ^2^ value of 0.9468 (Sukpancharoen et al., 2024). Characteristic importance analysis revealed that factors such as polymer concentration, applied voltage, and feed rate significantly affect the fiber diameter.Hierarchical Structure Graph Neural Networks (HS-GNN) have been employed to predict the mechanical properties of carbon nanostructures, achieving prediction errors of only 5%–10% (missing unit or context here, please clarify). This approach enables rapid prediction and high-throughput screening of nanostructures for enhanced material design.While machine learning models provide powerful tools for predicting the properties of nanofibers, there are still challenges in generalizing these models across different materials and applications. Further research is needed to refine these predictive capabilities and address the complexity of nanofiber behavior in diverse environments.

Dimensionless numbers are critical for predicting the performance of nanofibers, especially in their manufacturing process and resulting properties. These numbers, such as the Weber number, Reynolds number, and capillary number, help correlate manufacturing conditions with fiber morphology and mechanical properties, enabling the design of nanofiber scaffolds tailored to specific applications.Weber number: Indicates the relative importance of inertial forces compared to surface tension, affecting fiber diameter and bead formation during spinning (Ravishankar et al., 2019). Reynolds number: Reflects the flow state of the polymer solution and affects fiber alignment and homogeneity. Capillary number: Relates to the balance between viscosity and surface tension and affects fiber stability during production.Applications in Nanofiber ProductionCentrifugal Jet Spinning (CJS): Dimensionless numbers guide the optimization of CJS parameters to produce beadless, continuous fibers from a variety of polymers.Electrostatic spinning technique: The interaction of these numbers affects the morphology and properties of electrostatically spun nanofibers. For example, K0.5Na0.5NbO3 nanofibers, which exhibit piezoelectric properties, are influenced by these dimensionless numbers (Wang et al., 2015). Although dimensionless numbers provide a framework for predicting the properties of nanofibers, variations in polymer properties and environmental conditions can lead to unexpected results (Feng et al., 2024). Therefore, further empirical validation is required in various applications.

## 3 Types of electrospinning

### 3.1 Uniaxial electrospinning

**FIGURE 2:**
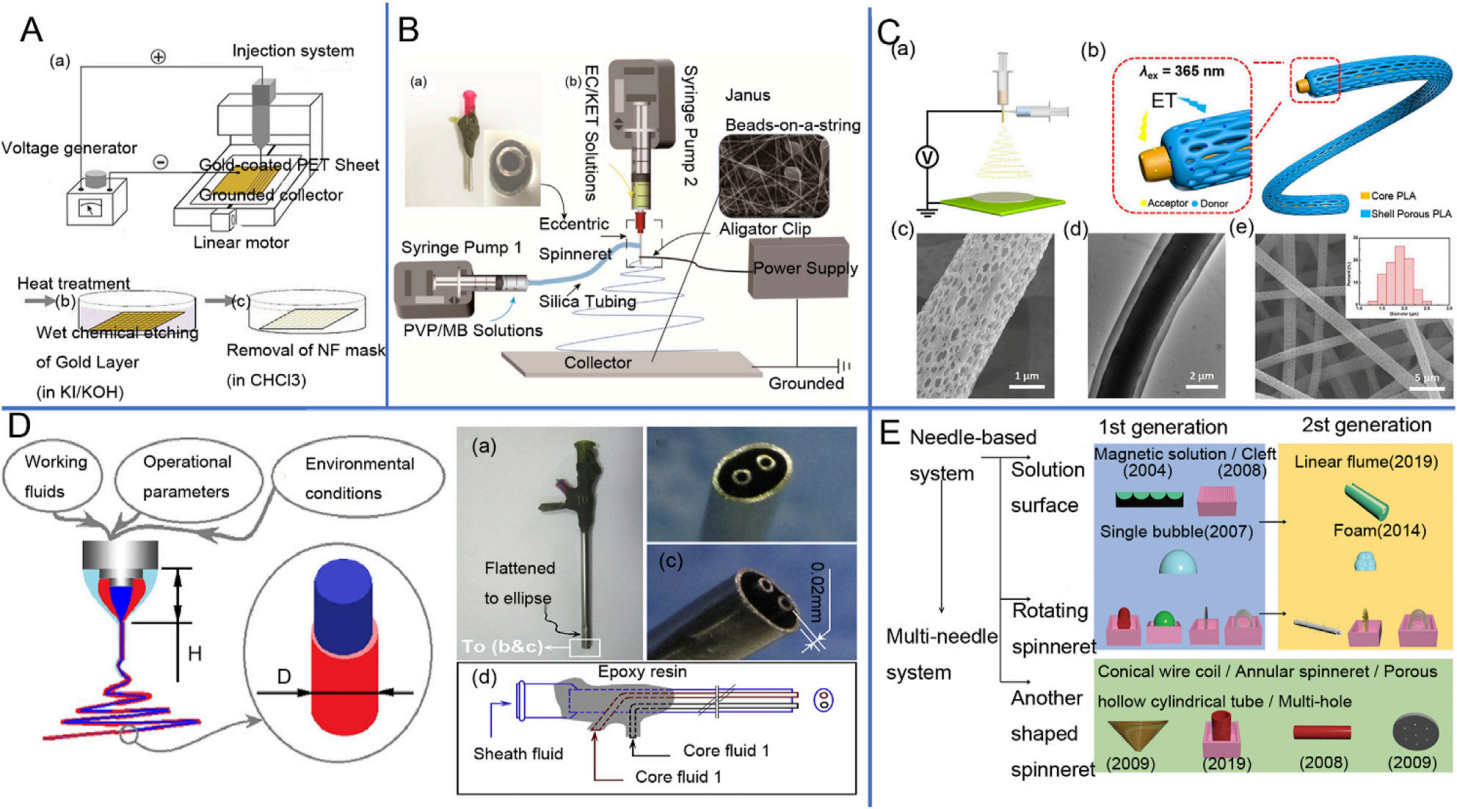
**(A)** Schematic diagram of single-axis electrostatic spinning device (Yousefi et al., 2019). **(B)** juxtaposed electrospinning device as well as the implementation process: (a) Image of a homemade eccentric spinneret; (b) Schematic of a coaxial homemade electrospinning system (Li D. et al., 2021). **(C)** Luminescent fiber membrane prepared by coaxial electrostatic spinning and electron microscopy images (Qin et al., 2018). **(D)** Schematic diagram of triaxial electrostatic spinning tip (Zhao et al., 2019), Design of a complex spinneret for the realization of three-fluid electrospinning: (a) digital image showing the full view of the spinneret; (b) front view; (c) side view; and (d) structural organization of the spouting from the three inlets67. **(E)** Schematic diagram of needleless electrospinning process classification (based on spinneret shape) and its generation (Raja et al., 2024).

Electrospinning can be divided into uniaxial electrospinning (Figure 2A), juxtaposedelectrospinning (Figure 2B), coaxial (Figure 2c), tri-axial (Figure 2D), multi-fluid (Figure 2E), and needless process (Figure 2F). The uniaxial electrospinning technique, introduced by Cooley and Morton in 1902, is the most commonly used method of electrospinning. Conventional uniaxial electrospinning involves a syringe filled with a polymer solution (Kang et al., 2020; Lv et al., 2025) and a single-tube needle. The needle, placed either horizontally or vertically, is stretched into filaments by a high-voltage electric field before finally falling onto a receiver (Gong et al., 2024a). Table 2 clearly illustrates the key advantages and disadvantages of uniaxial electrospinning, providing valuable insights into this innovative technology.

**TABLE 2:** Advantages and disadvantages of uniaxial electrospinning.

Advantage	Drawbacks	References
The setup is straightforward, accommodating a broad spectrum of polymers, and readily scalable	The process has limited control of fiber morphology and low production efficiency	Kızıldağ (2021)
It is possible to build tissue structures with specific orientations using stable and low-moving jets	The process faces several challenges, including low mechanical properties, very complex trajectories of polymer jets, limited technology, and a limited choice of solvents	Xu and Zhang (2017)
It controls fiber orientation, improves mechanical properties, and enhances material functionality	The material suffers from inadequate mechanical properties, hydrophobicity, a poorly controllable pore structure, and poor shrinkage and deformability	Senthamizhan et al. (2017)
An increased specific surface area can regulate fiber morphology and function. Efficient assembly of aligned nanofibers is possible	Limited control of optical fibers by uniaxial electrostatic spinning	Hanuman et al. (2023a)
An increased specific surface area can regulate fiber morphology and function. Efficient assembly of aligned nanofibers is possible	Difficulties in manufacturing 3D structures	Jana et al. (2012)

### 3.2 Coaxial electrospinning

Coaxial electrospinning involves the independent delivery of two different fluids to a coaxial needle, forming composite nanofibers with a core-shell inner structure (Sun et al., 2003; Zhou et al., 2024a), which is the most fundamental bi-compartment structure (Shi et al., 2024). The coaxial spinneret consists of a concentric arrangement of two needles (Lv et al., 2024; Kang et al., 2024), one thicker and one thinner. Its plane structure is similar to concentric circles (Gong et al., 2024b). As the core of the spinning head is slightly smaller than the shell of the spinning head (Chen et al., 2024c), the internal spinning head needle tip is slightly higher than the external spinning head needle tip. The two different streams of fluid have different flow rates at the needles (Lu et al., 2016; Chen et al., 2024b). Coaxial electrospinning is depicted in Figure 2. The limitations and advantages of the process are summarized in Table 3.

**TABLE 3:** Limitations and disadvantages of coaxial electrospinning.

Advantage	Limitations
The controllability of generated core-shell fibers is important (Mostofizadeh et al., 2023). Enhanced controllability of nanofiber membranes enables high-precision fiber deposition Core-shell nanofibers can be applied to composite reinforcement (McCann et al., 2005), Smart Textiles (Jiang et al., 2018), energy storage (Liu et al., 2020), and other applications	The coaxial electrostatic spinning process is complex and requires a special spinning head with two spinnerets that necessitate different process parameters. Additionally, coaxial needles are expensive, and cleaning the spinning head is laborious
The core-sheath structure delays the release of substances during drug delivery and prevents the explosive release of the drug at the initial stage. A drug delivery system can control specific drug concentrations. The rate of drug release can be controlled by the chemical properties of the core-shell structure’s surface (Braghirolli et al., 2014).	Achieving a balance between the flow rates of the two solutions is more difficult to accomplish. You need to control a variety of factors, including the different solvents’ surface tension, relative molecular weight, viscosity, electrical conductivity, and volatility. Failing to grasp these differences may result in a non-uniform composite fiber
Coaxial electrostatic spinning technology offers higher productivity compared to traditional single-needle heads, allowing two materials to be combined with greater controllability over the resulting fiber (Han and Steckl, 2019)	Both solutions need to be well-biocompatible and possess similar physicochemical properties

Coaxial electrospinning allows the production of highly diverse fibers, including core-shell structures, where each component maintains its individual material properties. By taking advantage of this property, complex materials can be fused into a single composite fiber based on their properties (Wang et al., 2010). Materials that are chemically or physically unstable, such as enzymes or rapidly degradable compounds, break down quickly when exposed to the external environment. Therefore, such compounds can be preserved by encapsulating them in the outer shell layer through coaxial electrospinning technology, where the shell layer serves as a protective barrier for these compounds. Coaxial electrospinning allows control over polymer properties and enables the development of biocompatible and mechanically stable materials, which has led to widespread interest in the biomedical field (Han et al., 2008). Coaxial electrospinning (CES) is a valuable technology in the field of drug delivery, with a wide range of applications. Through this technology, it is possible to produce nanofibers with superior performance compared to uniaxial electrospinning (Keirouz et al., 2020).

### 3.3 Side-by-side electrospinning

In recent years, juxtaposed electrospinning technology has seen rapid development in the preparation of multifunctional nanofibers and has garnered widespread attention from researchers (Zhou et al., 2024b). Especially in the fields of drug delivery and photocatalysis, not too long ago, Bligh and others (Annie Bligh et al., 2023) developed a multilayer nanofiber with a juxtaposed structure to serve as a multidrug carrier. This was achieved by using a three-stream juxtaposition electrospinning technique to prepare three-layer Janus fibers (Sun et al., 2024; Chen et al., 2023), designing a suitable eccentric juxtaposition nozzle, and selecting a suitable spinnable solution. The successful preparation of PVP/PVPV-CA/CA three-layer Janus fibers was achieved through three-fluid parallel electrospinning (Wang W. et al., 2025). With a smooth surface and a pronounced internal structure that demonstrates great potential in drug delivery (Zhang S. et al., 2024; Bibi et al., 2025), this fiber provides a flexible platform for multi-drug delivery while expanding the choice of materials. Meanwhile, Janus fibers prepared by juxtaposed electrospinning technology have unique advantages in terms of performance enhancement compared to single, core-shell, and structured fibers. Additionally, a study by Chang et al., in 2019 used TiO_2_/CoO juxtaposed nanofibers for photocatalytic degradation experiments of rhodamine B. By employing a simple single-spinning method, they successfully prepared juxtaposed nanofibers with relatively charged nozzles. These nanofibers exhibit excellent morphological, structural, and optical properties, thus demonstrating excellent photocatalytic activity and higher catalytic efficiency compared to single composite fibers (Chang et al., 2019). In a study by Liu et al., two-component nanofibers containing polyaniline were prepared using a juxtaposed electrospinning technique. This technique utilized camphor-doped polyaniline and polyethylene glycol, prepared with a special spinneret. The resulting juxtaposed nanofibers exhibited higher electrical conductivity and improved mechanical properties, as well as better toughness and resistivity, demonstrating their potential for application in the field of flexible sensors (Liu et al., 2019). The above studies demonstrate the growing applications and technological innovations of juxtaposed electrospinning technology in the fields of biomedical engineering and the environment.

### 3.4 Multiple-fluid electrospinning

Multi-fluid electrospinning is a new branch of electrospinning nanofiber preparation technology (Zhang et al., 2023b), which generates nanofibers with complex structures by using two or more liquid materials. The core technology of multi-fluid electrospinning lies in the combination of many different fluids, allowing different materials within the same nanofiber to exhibit various functions and properties. Multi-fluid electrospinning achieves functions and performances that single-fluid electrospinning cannot. Through multi-fluid electrospinning, it is possible to create coaxial fibers, core-shell structure fibers, or hierarchical fibers. This complex structure of the fiber has enhanced functionality (Xu et al., 2024), such as mechanical strength, electrical conductivity, and chemical properties, all within a single material.

Multifluid electrospinning, as an advanced technology for producing complex nanofibers, plays a vital role in the development of innovative sensors. This technology enables the production of nanofibers with unique properties, which can be used to manufacture a variety of high-performance sensors. Electrospun nanofibers can serve as electrochemical sensors to monitor environmental pollutants. Sensors utilizing electrospun multifluidic technology are not only sensitive but also highly scalable in their monitoring methods (Du et al., 2022). In addition, wet electrospinning of polyvinylidene fluoride (PVDF) combined with PEDOT can be used to prepare pluripotent piezoelectric nanofibers with high sensitivity and strain-sensing capabilities. These nanofibers can be applied to wearable electronic devices, especially in applications requiring flexibility and mechanical strain sensing, such as flexible electronic devices and industrial mechanical sensors (Hassanin et al., 2023). Nanofiber sensors containing dispersed magnetic particles, sodium alginate, and chitosan, prepared by multifluid electrospinning (Wang et al., 2020), are used in human-computer interaction, especially for wearable and flexible electronic devices. These sensors can provide high-precision signal recognition and are suitable for application scenarios requiring long-term stable use (Fu et al., 2024).

Multi-fluid electrospinning technologies, such as triaxial electrospinning, four-fluid electrospinning, coaxial electrospinning, etc., enable the direct spinning of different fluids into complex nanostructures. These advanced electrospinning technologies can then be post-processed for further optimization (Zhao et al., 2024). Their extensive application in the biomedical field, the facilitation of drug delivery systems, and implantable materials, provides a new way of thinking about next-generation functional sensors and nanomaterials (Yu et al., 2016; Yu et al., 2025).

### 3.5 Needleless electrospinning

Needleless electrospinning is a technology for manufacturing nanofibers. Unlike traditional needle electrospinning, it does not rely on a single needle to form nanofibers. Instead, it uses a free surface—such as a rotating disc, twisted wire, or gear-shaped rotator—to generate multiple polymer droplets. Therefore, needleless electrospinning can achieve multiple points of injection, generating fibers at multiple points. This can improve fiber efficiency and yield, and significantly increase the production efficiency of nanofibers, especially when using rotating discs or twisted metal wires as spinning nozzles (Holopainen et al., 2014). Needle-free electrospinning is a multi-point, multi-jet spinning process that forms multiple Taylor cones by applying a high voltage to a polymer solution or the surface of a melt, which then stretch to form nanofibers under the action of an electric field (Niu and Lin, 2012). Needle-free electrospinning devices typically include rotating discs, wires, or gear-shaped spinning nozzles, which are designed to produce fibers over a larger area and to reduce nozzle clogging problems (Huang et al., 2007). The innovative design of needleless electrospinning enables efficient production of nanofibers, overcoming the disadvantages and limitations of conventional needle-type electrospinning in terms of throughput and equipment maintenance. Needleless electrospinning can handle a wide range of polymer materials and different solution concentrations, making it potentially suitable for a wide range of applications in the fields of nanomaterials, medical devices, and textile manufacturing (Yalcinkaya et al., 2016).

Needle-free electrospinning has a wide range of applications in many fields due to its high efficiency and versatility. The nanofibers prepared by this technology have a high specific surface area and a good void structure, making their application in the filtration field outstanding. They can be used in air filtration, liquid filtration, oil-water separation, and other scenarios, exhibiting very high filtration efficiency. Needle-free electrospun fibers made of materials such as polyamide (PA) and polyvinylidene fluoride (PVDF) can be used for industrial and domestic HEPA filters (Holopainen et al., 2014). In the biomedical field, nanofibers produced by needle-free electrospinning are widely used in tissue engineering, drug delivery, and wound dressings. This is due to the excellent biocompatibility of the materials used to make the nanofibers, which can be used as scaffolding materials to support cell growth. Studies have shown that needle-free nanofibers made from polycaprolactone (PCL) and gelatin can provide the ideal environment for cells, favoring the regeneration of cartilage and skin tissue (Partheniadis et al., 2020). Because needleless electrospinning can produce fibers with a very high specific surface area, these fibers can be widely used in batteries and supercapacitors to increase their energy storage capacity. Carbon nanofibers prepared by electrospinning technology can be used as electrode materials for lithium batteries, enhancing the conductivity and energy density of the battery (Ramakrishnan et al., 2016). The porous structure of needleless electrospun nanofibers can also act as a catalyst carrier, increasing the reaction rate of catalysis, which plays an important role in water treatment and air purification (Tang et al., 2010).

Needle-free electrospinning technology has broad and extensive application prospects in many fields due to its high efficiency and convenience. It not only improves the efficiency of material production but also provides unique properties that differ from those of traditional nanofibers. These properties hold significant practical value in the fields of healthcare, biomedicine, energy, and the environment.

## 4 Types of electrospun nanofibers

### 4.1 Inorganic nanofiber

**FIGURE 3:**
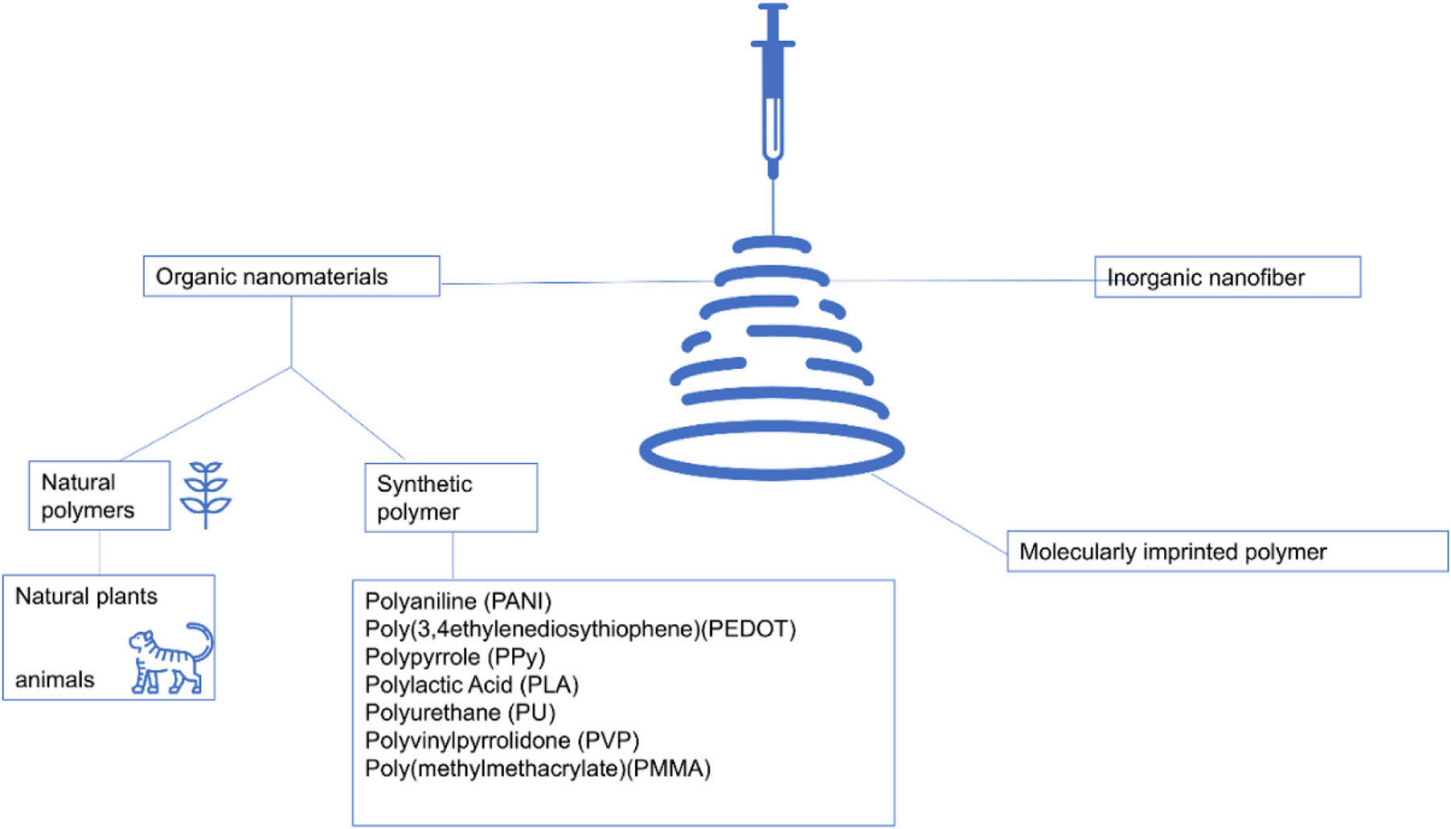
Electrospinning encompasses a diverse array of nanofiber categories, primarily categorized into inorganic, organic, and hybrid organic-inorganic nanofibers (Ding et al., 2019; Zhang et al., 2024c).

Nanofiberscan be categorized into inorganic, organic, and hybrid organic-inorganicnanofibers (Figure 3). Inorganic materials often have their special functional properties (Peng et al., 2024; Fan et al., 2024). Inorganic nanofibrous materials can further expand their functional applications. Such materials can be prepared using electrospinning techniques with polymers and metal precursors, resulting in nanofibers with high surface area-to-volume ratios (Toncell, 2021). Inorganic nanofiber materials can be synthesized by dispersing inorganic nanoparticles in a solvent at specific ratios using electrospinning technology, which can enhance mechanical stability and heat resistance. Flexible inorganic nanofiber yarns can be obtained through the electrospinning process, expanding the applications of inorganic nanofiber materials through a simple preparation method. Binary inorganic nanofibers, composited with graphene oxide and various nanomaterials, were experimentally analyzed and found to have excellent mechanical properties, electrical conductivity, and photocatalytic properties.

Inorganic nanofiber materials are versatile and can be added to composites to enhance their mechanical and thermal properties (Elfaleh et al., 2023; Salih and Kadhim, 2023). They are also used as antimicrobial agents in medical and filtration environments (Wen et al., 2022). They are also utilized in the process of tie-dyeing fabrics (Toncelli, 2021). The electrospinning technique allows the design of polymer nanofibers with high surface area-to-volume ratios, thus broadening the range of applications to multifunctional compositions. Flexible inorganic nanofiber yarns, prepared by the electrospinning technique, provide a new method for the preparation of highly bendable pure inorganic nanofiber structures, broadening the range of materials available for inorganic nanofibers. Electrospun inorganic nanofiber materials have the potential for versatility and full development of utilization in various fields.

### 4.2 Organic nanomaterials

#### 4.2.1 Natural polymers

Natural polymers are polymer compounds derived from the basic structures of natural plants and animals, known for their good biocompatibility and biodegradability. Plant-derived polymers primarily consist of substances such as sodium alginate, plant-based proteins, cellulose, lignin, natural rubber, and starch. Cellulose nanofibers possess excellent properties that make them ideal for green products and polymer composites, including jute, flax, ramie, hemp, cotton, and sisal. These fabric fibers are hydrophilic due to their hydroxyl groups (Elfaleh et al., 2023). Animal-derived polymers encompass collagen, chitosan, chitin, and gelatin as their primary constituents. Chitosan (CS) is a naturally occurring polysaccharide derivative known for its good biocompatibility, biodegradability, and amino activity (Abraham et al., 2020). CS composite nanofibers can be used in wound dressings, where they aid in wound healing by mimicking the extracellular matrix (Manasa et al., 2023). However, due to its high molecular weight and low solubility, chitosan is difficult to spin directly into filaments. Blending chitosan with other polymers can enhance its spinnability, thereby improving the morphology and quality of the resulting nanofibers. Chitosan-collagen composite nanofiber mats embedded with curcumin were prepared by a single-step electrospinning process and can be applied to burn patches. The combination of chitosan and collagen maintains long-term stability and optimal physicochemical properties, enabling better cell adhesion and proliferation. Additionally, curcumin can be added to enhance its antimicrobial capacity (Castellano et al., 2023). In addition, post-modification techniques are used to functionalize chitosan nanofibers with different chemical compositions, making them suitable for various fields such as tissue engineering, drug delivery, and sensors (Welchoff et al., 2023). Chitosan has also attracted significant attention in the field of electrochemistry. CS can be combined with various nanomaterials to significantly improve electrochemical detection capabilities, primarily including carbon nanomaterials, metal nanoparticles, and conductive polymers. Molecularly imprinted sensors, when combined with chitosan, exhibit excellent sensitivity, selectivity, and stability, enabling accurate and reliable detection of drugs (Bounegru and Bounegru, 2023).

#### 4.2.2 Synthetic polymer

Synthetic polymers are artificially synthesized and possess unique properties. They are widely used in electrospinning, a technique now favored by researchers (Song et al., 2024). The characteristics of some artificial polymers and their applications are shown in Table 4.

**TABLE 4:** Characteristics and applications of synthetic polymers.

Synthetic	Characteristic	Application	References
Polyaniline (PANI)	The distinctive π-π conjugated framework serves as a mediator for electron exchange between the redox-active site and the enzymatic electrode, thereby enhancing the transfer of electrons to the electrode surface	A graphite rod (GR) electrode is equipped with a glucose biosensor that incorporates a conductive polymer nanocomposite fiber, which is impregnated with glucose oxidase (GOx) and adorned with gold nanoparticles	German et al., 2020; Neupane et al., 2021; Popov et al., 2021
Poly (3,4-ethylenediosythiophene) (PEDOT)	The substance demonstrates remarkable electrochemical performance, including efficient ion and electron mobility, elevated conductivity, and robust stability. It also boasts exceptional physicochemical inertness, favorable biocompatibility, along with desirable reversibility and consistent reproducibility	An unlabeled glucose sensor for electrochemical detection has been fabricated from polystyrene sulfonate (PSS), titanium carbide (Ti3C2), and graphene quantum dots (GQDs)	Zahed et al., 2020; Kitova et al., 2021; Nashruddin et al., 2021
Polypyrrole (PPy)	The substance is characterized by its non-toxicity, high electrical conductivity, porous architecture, distinctive molecular identification mechanism, and favorable biocompatibility	A graphite electrode-based electrochemical glucose sensor, enhanced with nickel nanoparticle and polypyrrole (PPy) composite modifications	Emir et al., 2021; German et al., 2021
Polylactic Acid (PLA)	Thermoplastic polyester, extracted from starch and rice, is a renewable source of composite materials that are easy to form. These materials offer simple processing and recyclability, along with mechanical, thermal, and rheological properties	Medical, food packaging, textile, and many other industries	Paul et al. (2023)
Polyurethane (PU)	The growth and polymerization of the material occur via the interaction of three fundamental components: the isocyanate group, polyol compounds, and chain-extending agents of low molecular weight	Innovative glucose detection strips utilize polyurethane-based hollow nanofiber matrices, fabricated through the process of co-axial electrospinning	Ji et al. (2014)
Polyvinylpyrrolidone (PVP)	The polymer is water soluble, chemically inert, amorphous, Capable of dissolving in aqueous and numerous organic mediums, it also exhibits minimal toxicity	Nitrogen-doped double-walled carbon nanotubes (N-DWCNTs) and PVP were mixed to create a highly sensitive and selective formaldehyde sensor, characterized by high sensitivity and selectivity	Chobsilp et al. (2022)
Poly (methylmethacrylate) (PMMA)	The non-water-soluble, amorphous hydrophobic polymer is readily dissolved in various organic solvents, demonstrating excellent chemical inertness and resistance to weathering		Simões et al. (2019)

### 4.3 Molecularly imprinted polymer

Molecularly imprinted polymers (MIPs) possess specific recognition capabilities for the selective adsorption of particular target molecules and their structural analogs. These materials are synthesized using molecular imprinting techniques (Jia et al., 2018). The technology mimics the specific interactions that occur in nature, such as those between enzymes and substrates, and between receptors and antibodies. MIPs are more thermally and chemically stable than other biorecognition primers (Pazur and Kleppe, 1964).

MIPs have been widely used in various types of urinary albumin sensors, and these sensors exhibit very high sensitivity in detecting urine samples, which is crucial for the prevention and early detection of kidney disease. The polymer hydrogel sensors prepared by MIP effectively adsorb proteins such as albumin. Biosensors that use MIPs have high affinity, low detection limits, and wide linear ranges for detecting complex biological fluids, including serum, saliva, cerebrospinal fluid, sweat, and urine. Utilizing MIPs, biosensors demonstrate high affinity and a wide linear range with a low detection limit for complex biological fluids (Mustafa et al., 2022). MIPs can enhance the selectivity and sensitivity of albumin detection in urine. Zhang et al. synthesized MIP using HAS as a template and phenylenediamine (o-PD) and p-diphenol (HQ) as functional monomers. They synthesized it by electropolymerization reaction and designed a molecularly imprinted electrochemical biosensor based on a dual-signaling strategy for the detection of human serum proteins. This biosensor possesses low detection limits, good selectivity, and biocompatibility (Zhang et al., 2019). Resende et al. fabricated a novel label-free sensor for the detection of fibrinopeptide B (FPB) in urine, which can help prevent venous thromboembolism. They obtained molecularly imprinted photonic polymers (MIPPs) by vertically depositing highly ordered silica nanoparticles tailored to MIPs. The sensor is capable of rapidly and sensitively detecting FPB in pairs of urinary samples and can be used for point-of-care testing of various protein markers (Resende et al., 2020).

Molecularly imprinted polymers (MIPs) serve as an additional means of analysis, complementing traditional enzyme-based devices. The synthesis and optimization of MIPs, along with the use of polydimethylsiloxane (PDMS) pressed into a polyvinyl chloride (PVC) adhesive layer, create opportunities for non-invasive glucose sensor applications with a wide linear range (Caldara et al., 2021).

## 5 Applications in sensors for healthcare

Electrospinning can produce nanofibers with unique properties, making sensors made by this technology useful in various aspects of life. Various types of sensors can be produced, such as pressure sensors, temperature sensors, humidity sensors, gas sensors, biochemical sensors, and biosensors. Today, the world of physical sensors in biomedicine, tissue engineering, and other fields has made significant progress. Electrospinning technology is one of the important preparation methods, and compared to other technologies, it offers advantages such as nanoscale effects, high specific surface area, high porosity, and more (Cao et al., 2023).

### 5.1 Pressure sensors

**FIGURE 4:**
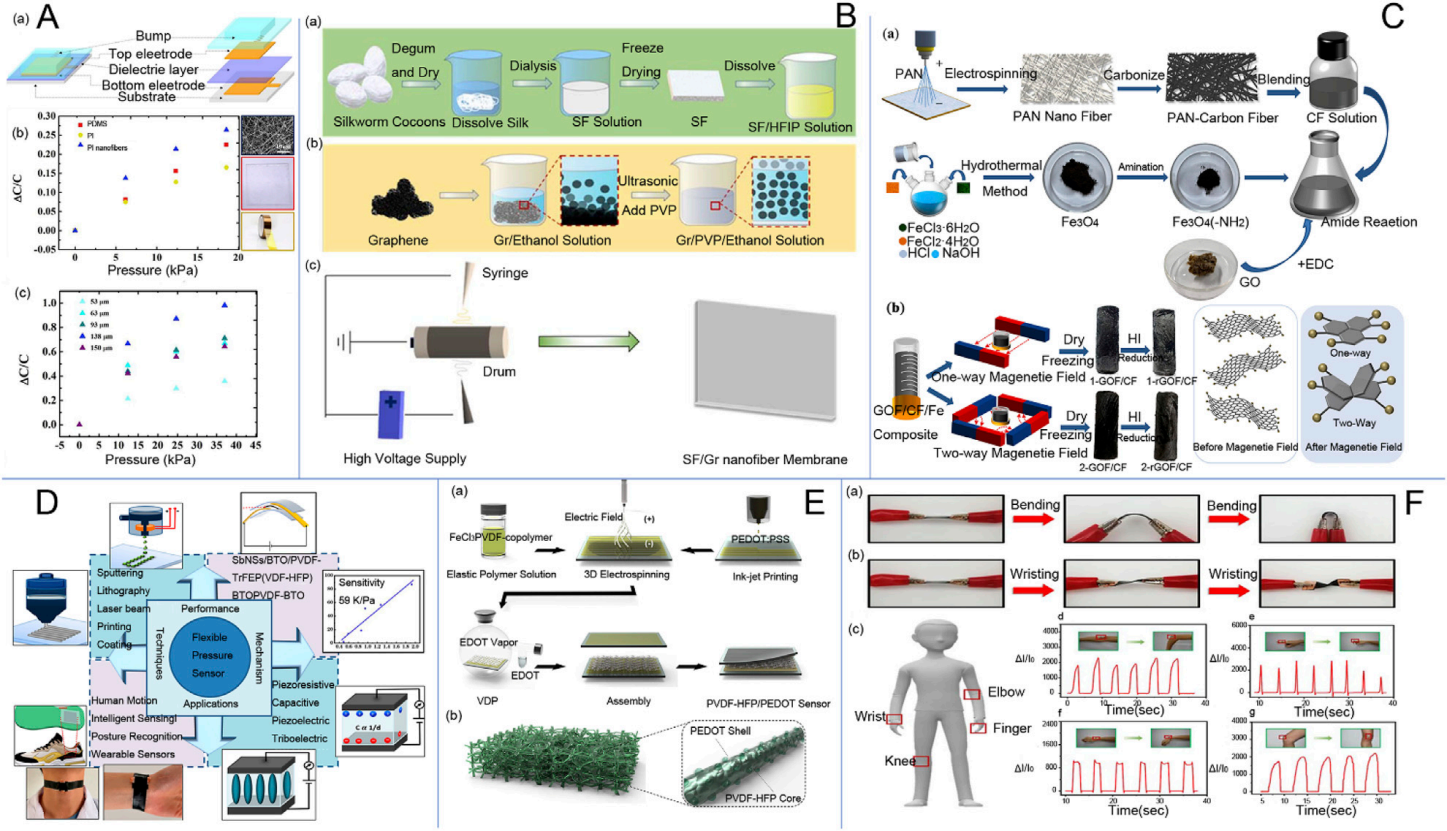
**(A)** (a) Structural diagram of the capacitive pressure sensor; (b) Relationship between capacitance change and pressure when PI nanofiber membrane, PDMS, and PI tape were used as the dielectric layers, respectively; and (c) Relationship between capacitance change and pressure when PI nanofiber membrane with different thicknesses (53 μm, 63 μm, 93 μm, 138 μm, and 150 μm) was used as the dielectric layer (Zhu Y. et al., 2020). **(B)** (a) Extraction process of silk fibroin; (b) Preparation of graphene/polyvinylpyrrolidone/ethanol (Gr/PVP/ethanol) solution; (c) Schematic diagram of (silk fibroin/graphene) (SF/Gr) nanofibrous membranes prepared by electrospinning (Liu et al., 2022). **(C)** Graphene/electrospun carbon nanofiber sponge composites induced by magnetic particles for multi-functional pressure sensor (Ai et al., 2023), (a) Preparation process of rGOF/CF composite sponge material. (b) Schematic of the magnetic field sensing process of rGOF/CF sponge material. **(D)** current status of research on flexible pressure sensors (Naz et al., 2024). **(E)** Schematic diagram of the experimental setup and PVDF-HFP/PEDOT pads (Kweon et al., 2018). a Schematic of the fabrication process of PVDF-HFP/PEDOT NF-based piezoresistive pressure sensors and b Schematic of the electrospun 3D nanostructured PVDF-HFP/PEDOT NF pads. **(F)** SF/Gr Flexible Pressure Sensors for Real-Time Monitoring of Human Activity and Detection of Physical Signals.

Pressure sensors play a role in monitoring and controlling processes, thus holding an important role in the field of electrospinning research. Consequently, various types of pressure sensors have been developed, such as capacitive pressure sensors, piezoelectric pressure sensors, and flexible pressure sensors. Figure 4A shows a flexible capacitive pressure sensor based on electrospun polyimide (PI) nanofiber membrane as a dielectric layer. Capacitive pressure sensors with nano-ionized membranes offer high sensitivity and a wide measuring range, enabling them to be applied for precise pressure monitoring. Piezoelectric pressure sensors are flexible and durable, making them suitable for monitoring human movement. Additionally, flexible pressure sensors based on electrospinning technology are self-powered, breathable, and stretchable. They can be integrated into electronic smart wearable devices, leveraging the nanofibers prepared by electrospinning. Massaglia and Quaglio (2023) A stretchable, piezoresistive flexible sensor design was obtained using the electrospinning technique for integration into electronic voucher platforms. Figure 4B is a nanofiber membrane prepared by double electrospinning method. This demonstrated the good performance of the new nanofiber pressure sensor. Electrospinning technology allows the fabrication of piezoelectric microfiber sensors with simple structures and high flexibility, making them suitable for sensing weak mechanical excitations. Jiang et al. (2023) fabricated microfibers of piezoelectric poly (vinylidene fluoride) on a substrate of flexible poly (ethylene terephthalate) using electrospinning. Experiments demonstrated that the open-circuit voltage response of the piezoelectric microfibers was strain-dependent and that the microfibers could detect sound signals recorded between 70 and 120 dB, with the sound frequency aligning exactly with the nominal frequency. Additionally, the slight wind noise from a hand-cranked fan can also be detected. This fabrication method offers a promising, low-cost approach for sensing weak mechanical excitations. Piezoelectric polyvinylidene fluoride (PVDF) nanofibers with a bi-oriented structure introduce piezoelectric anisotropic BaTi2O5 nanorods to construct flexible piezoelectric energy harvesters (PEHs). These PEHs can be utilized for multimodal smart biomonitoring of the human body and are suitable for wearable piezoelectric pressure sensors (Shao et al., 2023). In addition, Kong et al. (2023) fabricated a composite membrane of poly (vinylidene fluoride) (PVDF) and barium titanate (BaTiO3) by in-field electrospinning. After drying, the layered structured membrane was obtained. Different membranes were then combined with different substrates to study the effect of the encapsulation scheme on the sensors. The conclusions show that the layered structured membrane and layered structured substrate (HHS) piezoelectric sensors can be used to monitor human movement, indicating the great potential of layered microstructured flexible piezoelectric pressure sensors for human activity monitoring and wearable biological devices. Flexible pressure sensors are an important component of soft electronics due to their tactile sensing capabilities (Ren et al., 2022). Zhou et al. (2022) obtained an ultra-thin, flexible pressure sensor with ultra-high sensitivity by controlling the electrospinning time. This technology allows for the creation of devices with different sensing characteristics and has been studied. Tactile sensors used in the robotics industry can also be manufactured using electrospinning technology. Additionally, pressure sensors made from low-cost random polystyrene as the base polymer can be used in robotic arms to simulate the sense of touch (Ramadoss et al., 2021). Polyvinylidene fluoride (PVDF) and PVDF/TiO2 are prepared by electrospinning to enhance the piezoelectric effect. This effect is closely related to the capacitance characteristics of the sandwich structure in capacitive sensors, which consist of EVA foam as a cover layer, conductive copper fabrics, and nanofibers. These sensors are lightweight, flexible, and suitable for use in wearable electronic devices. They can provide time-dependent changes in the magnitude of force, offering valuable information on the effect of applied forces (Daňová et al., 2020).

Flexible sensors represent a popular area of research within the field of electrospinning technology. Figure 4C shows an innovative preparation of lightweight, highly sensitive three-dimensional (3D) conductive nanofiber sponges for pressure sensors. These highly sensitive pressure sensors are widely used in electronic skin, human physiological monitoring, and artificial intelligence (Zhi et al., 2023). Silk cellulose/graphene nanofiber membranes were prepared by the double-needle electrospinning method (Liu et al., 2022). Then, by superimposing a single-layer silk fibroin/graphene nanofiber membrane prepared by double-needle electrospinning, a three-dimensional composite hierarchical structure with high sensitivity was obtained. This structure was encapsulated using poly (dimethylsiloxane) (PDMS), resulting in pressure sensors with good durability and short response times. Wearable devices require real-time applicability, but complex steps and reliable durability often limit their application. To address this, researchers have proposed a solution-processable electrospun patterning candidate material scalable by electrospinning technology. This material is capable of forming ultra-long, mechanically robust nano-microfibers with more uniform bending durability (Veeramuthu et al., 2022). Stretchable and breathable skin-like pressure sensors with high sensitivity and all-weather antimicrobial activity were prepared by integrating silver phosphate nanoparticle-modified halloysite nanotubes (Hal@Ag_3_PO_4_) into thermoplastic polyurethanes (TPUs) via electrospinning. These sensors have high sensitivity, fast response times, low detection limits, good stability, and excellent breathability. Importantly, they are capable of providing all-weather antimicrobial activity against *Escherichia coli* and *Staphylococcus aureus* (Figure 4D). This enables real-time, accurate, and continuous detection of external stimuli and physiological signals in wearable sensor devices. With all-weather antimicrobial activity, the flexible pressure sensor can achieve real-time, accurate, and continuous detection and monitoring of external stimuli and physiological signals in wearable sensor devices (Wang et al., 2022). A flexible and highly sensitive pressure sensor composed of iron oxide (Fe_3_O_4_) and carbon nanofibers (FeOCN) was fabricated using 3D electrospinning and heat treatment to accurately detect wrist pulse, articulation, respiration, and finger flexion for healthcare applications (Cai et al., 2021). Figure 4E exhibits the electrospun three-dimensional membranes as piezoresistive sensors. The sensor, based on a PVDF nanofiber membrane prepared by electrospinning technology, has good piezoelectric characteristics and enables quantitative pressure measurement. It can be used as a real-time monitor of human body movement. Additionally, this sensor realizes non-contact sensing, which provides a broad idea for the future of non-contact human-computer interaction (Wang et al., 2018). Pressure sensors based on microstructured electrospun rough polyurethane (PU) nanofiber membrane assembly have been developed (Xue et al., 2022). The microstructure features a rough upper surface and a smooth lower surface. The introduction of a silver conductive layer on the surface of the polyurethane film layer enables the polyurethane/silver film to function as a piezoelectric sensor. Sensors with a spacer layer, fabricated using near-field electrospinning technology, utilize a polyurethane fiber base as the spacer layer at the interface between the piezoresistive layer and the contact electrode. This material plays an important role in optimizing sensors and improving their sensing range and sensitivity. Hybrid structures of organic and inorganic materials are also used in the production of flexible sensors using electrospinning technology. Electrospun tetragonal barium titanate (BaTiO_3_) and polyvinylidene difluoride (PVDF) composite materials can enhance pressure sensitivity. In addition, composite fibers consisting of multilayers for stretchable pressure sensors offer higher sensitivity compared to pure PVDF sensors (Kalani et al., 2020). Electrospinning technology is a prominent method for fabricating flexible pressure sensors, capable of producing nanomaterials with high sensitivity and a wide linear range.

### 5.2 Wearable sensors

Most of the energy supply devices for sensor devices in the wearable field are made of hard and bulky materials, making them difficult to wear. As wearable devices continue to evolve in various fields, many sensors are now used to detect human physiological indicators. However, their resistance to the environment, sensitivity, and mechanical properties are not yet at the desired level. The excellent properties of nanofibers and nanofiber membranes prepared by electrospinning are able to satisfy the needs of sensors for wearable devices. The working principle of biosensors involves the addition of substances embodying the physiological indicators of humans, such as urine, sweat, and blood, which are converted into electrical signals.

**FIGURE 5:**
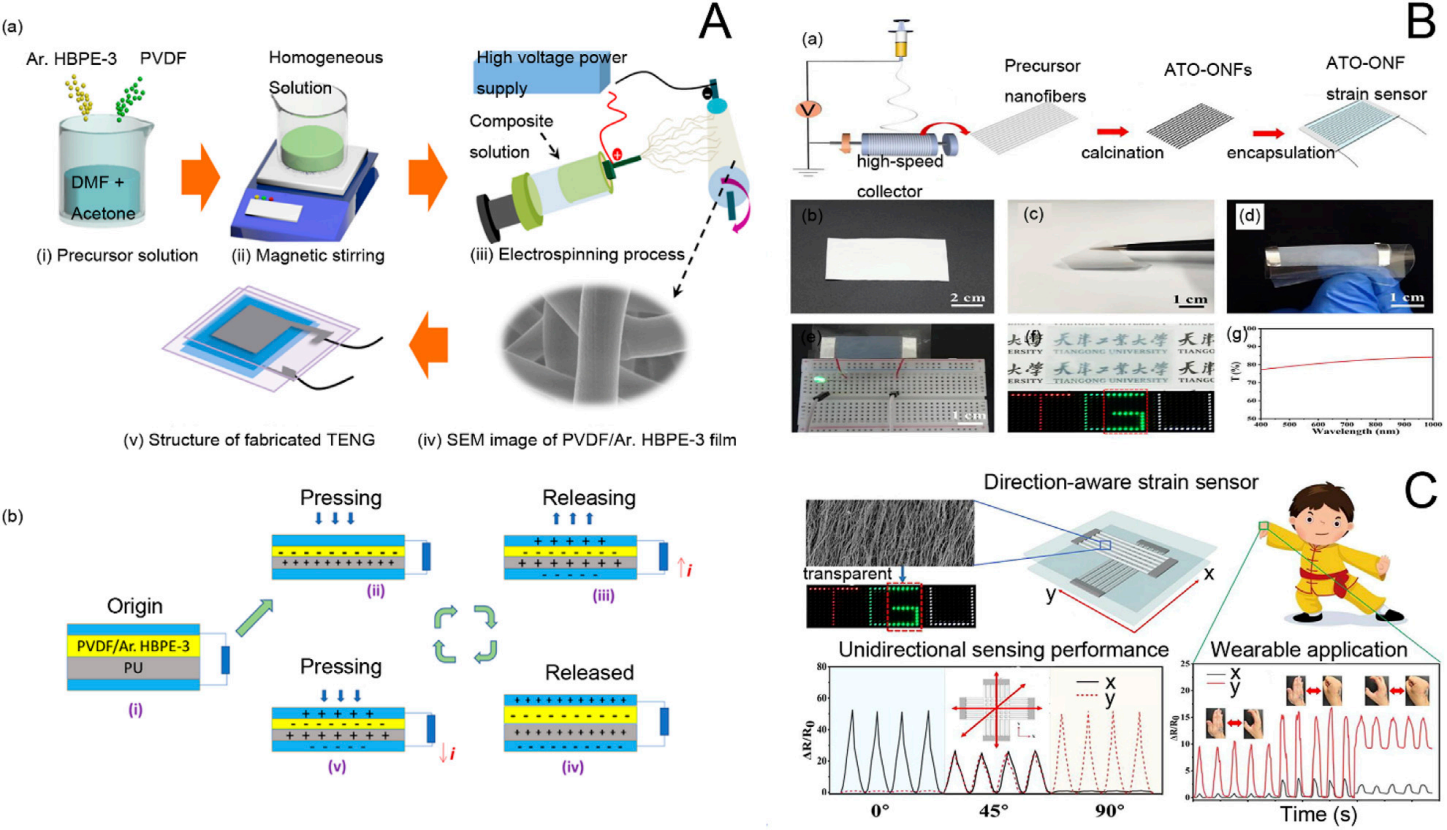
**(A)** (a) Schematic representation of the preparation of P-Ar.HBP-3 solution and electrospinning and fabrication of TENG: (i). HBP-3 mixed solution preparation, (ii). Mixed solution by constant magnetic stirring, (iii). Scanning electron microscope images of electrospun nanofibers, (v). Manufactured TENG equipment. (b) Schematic diagram of the TENG mechanism (Gunasekhar et al., 2023). **(B)** Fabrication of single-layer ATO-ONF strain sensors. (a) Schematic of the fabrication process of a single-layer ATO-ONF strain sensor. (b) Photograph of the oriented precursor nanofiber film. (c) Photograph showing the flexibility of the ATO-ONF film. (d) Flexible ATO-ONF strain sensor under bending. (e) Conductivity demonstration, (f) transparency demonstration, and (g) transmittance spectrum of the ATO-ONF strain sensor. **(C)** Oriented Electrostatically Spun Nanofibers for Highly Sensitive, Orientation-Aware, and Transparent Strain Sensor Applications (Yang et al., 2022).

For example, Mohammadpour-Haratbar et al. (2022) investigated a bimetallic nanomaterial sensor with electrospun carbon nanomaterials pure/oxidized Ni, Co for accurate detection of glucose with good selectivity for human serum proteins and no interference with interfering oxides such as ascorbic acid (AA), uric acid (UA), and dopamine. Kweon et al. (2018) prepared conductive core/sheath chitosan nanofibers consisting of (polyvinylidene fluoride-hexafluoropropylene) (PVDF-HFP)/poly (3,4-ethylenedioxythiophene) (PEDOT) by electrospinning for the fabrication of piezoresistive pressure sensors. These sensors can be used in wearable bracelets for monitoring blood pressure and open the way for the mass production of highly mechanical polymer pressure sensors. Wearable sensors use energy generated by pressure or mechanical deformation of materials. They can be used to detect the health status of the human body through heartbeat or pulse rate (Zhang et al., 2017). These sensors can be placed on the chest, nose, wrist, and fingers (Lee and Yun, 2017) with the other end connected to an electronic device such as a cell phone or a display to observe heart rate fluctuations. As technology evolves, these types of sensors are generally connected to smartwatches. Shao et al. (2023) constructed flexible piezoelectric energy harvesters (PEHs) by using piezoelectric poly (vinylidene fluoride) with a bi-oriented structure to introduce nanorods of barium titanate with piezoelectric anisotropy. This sensor can recognize forces in different bending directions and is a self-powered pressure sensor that can be used for intelligent biomonitoring of multimodal human movement. Gunasekhar et al. developed polyvinylidene fluoride/aromatic hyperbranched polyester-based third-generation electrospun nanofibers as nanogenerators for wearable devices for energy harvesting and health detection applications (Gunasekhar et al., 2023). The production process and working principle are shown in Figure 5A below. Schematic diagrams of the production of electrospun nanofibers and triboelectric nanogenerators (TENGs) for energy harvesting and health detection are shown in Figures 5i–iv. Figure 5i shows the preparation process of the solution of the precursor polymers. PVDF and Ar.HBP-3 (0–40 wt%) is dissolved in a solvent mixture of DMF: acetone (3:2 v/v). Then, the polymer solution is electrospun onto the substrate (Figure 5iii). The nano nets are stripped and used to further fabricate the TENG. FE-SEM imaging of the electrospun fibers is shown in Figure 5iv, and the structure of the TENG electrodes is schematically shown in Figure 5v. Figure 5B shows the working mechanism of TENG as shown in the sequence of Figures 5i–v. Before an external pressure is applied, the two films are isolated from each other without charge. After an external force is applied and the upper and lower layers are in contact, the more different electron affinities of the upper and lower layers cause the contact layers to form equal and opposite charges according to the principle of triboelectric effect. The balance of the charges in Figure 5b(ii) organizes the flow of the electric current, so that at the beginning of the release of the external load, the films slowly start to separate from each other, leading to a potential difference between the electrodes. To equilibrate the charges in Figure 5b(iii), electrons migrate from the top to the bottom, and the cycle is repeated until the charge equilibrium shown in Figure 5b(iv) is reached, due to the principle of electrostatic induction. The electrodes and the associated triboelectric film acquire the same amount of opposite charge. If the external force is increased, the electrostatic induction equilibrium will be out of balance, as shown in Figure 5b(v). The morphology produced in this paper alters a simpler and cleaner technique for manufacturing high-performance TENGs and suitable for serving as a potent energy generator in the context of mechanical energy recovery systems and wearable technology.

On the other hand, in personal healthcare monitoring, there is an urgent need for multifunctional electronic components that are highly adaptable, sensitive, long-lasting, and do not require external energy sources. For this reason, Cao et al. (2023) investigated a stretchable, skin-like, self-powered haptic and motion sensor based on a single-electrode mode triboelectric nanogenerator. With excellent sensitivity for recognizing low to high pressures (Figure 5C), this sensor can also be used for a wide range of human motion monitoring, offering great potential for future applications of skin-like surface electronics in medical monitoring.

### 5.3 Glucose sensor

**FIGURE 6:**
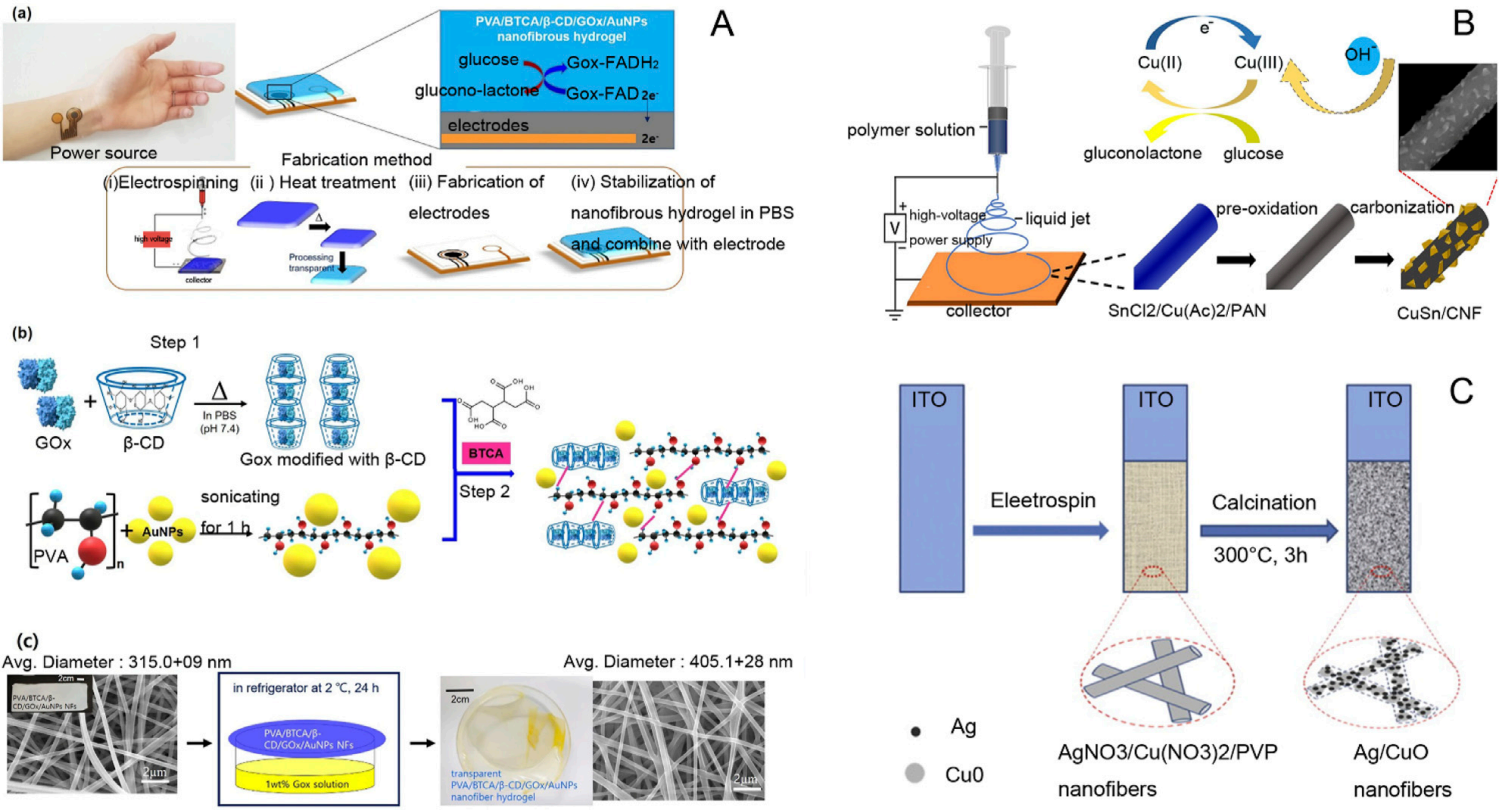
**(A)** (a) Schematic of a patch-type glucose sensor using PVA/BTCA/β-CD/GOx/AuNPs NF hydrogel on an electrode and a glucose sensing mechanism for noninvasive real-time monitoring of glucose in sweat. (b) Schematic of the preparation of PVA/BTCA/β-CD/GOx/AuNPs complex doping solution for electrospinning. (c) Preparation of transparent PVA/BTCA/β-CD/GOx/AuNPs nanofiber hydrogels (Kim and Kim, 2020). **(B)** Schematic diagram of electrochemical sensors prepared by composite controlled electrospinning of copper-antimony bimetallic nanoparticles/carbon nanofibers (Huan et al., 2022). **(C)** Schematic diagram of AgNPs/CuO nanofiber non-enzymatic glucose sensor electrode (Zheng et al., 2014).

Electrospinning technology is widely used in the field of glucose sensors. Biocompatible materials prepared by this technology offer a new process for the development of glucose sensors. Nanofibers prepared by electrospinning have higher porosity and a high specific surface area, which can enhance the sensitivity of glucose sensors. Additionally, they can be used to develop new enzyme-free glucose sensors. A schematic diagram of the glucose tracer (Figure 6A), an electrochemical glucose tracer (Figure 6B) and a non-enzymatic glucose sensor electrode (Figure 6C) are included in Figure 6. These advancements play an important role in real-life applications for the prevention, diagnosis, and treatment of diabetes.

Glucose sensors for enzymatic electrochemical sensors are based on electrospun nanofiber membranes (ENFM) of conductive polymers with glucose oxidase (GOx) immobilized directly on the surface of nanofibers. The sensor has good stability over long periods, has a wide linear range, and can detect glucose concentrations from 0-25 mM with a response time of a few seconds (Puttananjegowda et al., 2020). Nanofibrous membranes obtained by mixing silicon carbide nanoparticles (SiCNPs) with conductive polymers (CPs) can enhance the binding of glucose oxidase (GOx) within the fibrous membranes, thus improving the sensitivity of the sensing electrodes. The glucose electrodes have good stability and can be preserved for long-term use (Puttananjegowda et al., 2021). Highly dispersed Ni/CoO nanofibers were prepared using the electrospinning method, and Ni/CoO could be uniformly dispersed on the surface of carbon nanofibers. The Ni/CoO nanofibers prepared by this method have excellent electrocatalytic activity with a low detection limit, enabling them to determine low glucose concentration samples (Mei et al., 2019). Electrospun polyvinyl alcohol (PVA) can make flexible enzyme electrodes; the electrode is modified by silver nanoparticles, and ferrocene (Fc) is used as a glucose oxidase (GOD) receptor to minimize the effect of oxygen. The nanosilver can increase the surface area of the electrode to improve the stability of the conductivity. The glucose sensor has a high degree of flexibility due to the role of ferrocene, high performance, and a simple manufacturing process (Wang et al., 2023).

The biggest problem with enzyme sensors is that enzymes are unstable, and enzymatic glucose sensors are inactivated at temperatures above 40°C and at high or low pH levels (German et al., 2021). Non-enzymatic glucose sensors detect glucose directly by electrochemical methods without the need for an enzyme as a biorecognition element. They operate through the principle of electrochemical oxidation and detect glucose concentration by measuring the current signal. The stability of the electrochemical sensor is enhanced by electrospraying gold nanoparticles and electrospinning polyacrylic acid/polyacrylonitrile nanofibers (PAA/PAN/AuNPs) to increase conductivity, thus enabling non-enzymatic determination of glucose (Ismail et al., 2021). Sun et al. used the electrospinning method in combination with heat treatment to prepare nanocomposites of NiCF and CF doped with a large number of Ni and NiO nanoparticles. These nanocomposites offer good analytical performance, low cost, simple preparation, and effective high sensitivity (Liu et al., 2009). This method does not cause chlorine ion poisoning. The CuO-NiO nanocomposite fibers prepared by electrospinning, after high-temperature calcination to remove impurities, can better exhibit fiber properties. The electrode shows good selectivity, reproducibility, and long-term stability. A CuO-polyaniline nanofiber-modified fluorine-doped tin oxide (CuO/PANI-NF/FTO) electrode can be used as a non-enzymatic glucose sensor (Esmaeeli et al., 2018). The following table lists the materials, detection limits, detection ranges and sensitivities of some non-enzymatic glucose sensors are included in Table 5.

**TABLE 5:** Materials, detection limit, detection range, and sensitivity of non-enzymatic glucose sensors.

Makings	Linear range (mM)	Detection limit (μM)	Sensitivity (μA^-2^mM^-1^)	References
Pt	2.5–22			Park et al. (2012)
Au	0.01–10	4.13	291.6	Nugraha et al. (2017)
Pt-Au	0.2–4.8	1.3	12.85	Wang et al. (2016)
Ni	0.00025–1.2	0.01	0.0025	Darvishi et al. (2017)
NiO	0.001–0.27	0.033	1785.4	Cao et al. (2011)
Cu	0 8–11	0.59	3643	Guo et al. (2014)
CuO	0.0004–1.2	0.2	2596	Jiang and Zhang (2010)

The distinction between Non-Enzymatic and Enzymatic Glucose Sensors is multifaceted: a Non-Enzymatic Glucose Sensor operates without the need for enzymatic catalysis in glucose sensing. This type of sensor is straightforward to manufacture, simpler in construction, boasts a broad linear response, and ensures adequate specificity and reliability.

Electrostatic spinning is now a key technology for the development of sensors in various clinical areas, especially electrospun nanofibers (NFs). These nanofibers have a high surface area, high porosity, and high flexibility, which allows them to be used in a wide range of sensor applications, from biomarker detection to wearable detection devices (Haicka k.,and Cabaj., 2021).In the context of biomarkers, electrospun nanofibers can increase the sensitivity and selectivity in detecting disease biomarkers due to their high loading capacity and improved biomolecular activity (Zhou et al., 2024a). They have now been applied to a variety of diseases, including cancer and cardiovascular diseases, demonstrating the versatility of electrospun nanofibers in clinical diagnostics.In wearable health detection, electrospun nanofibers can be integrated into wearable sensors that provide real-time data about physiological signals (Angkawinitwong and Williams, 2021). These sensors can be used for continuous health monitoring. Wearable sensors are designed to accommodate dynamic tissues, overcoming the limitations of traditional sensors and thereby improving patient comfort and data accuracy (Wang and Zhang, 2024).In terms of pressure sensors, electrospun fiber membranes developed specifically for pressure sensors exhibit excellent stability and sensitivity for real-time physiological detection. Pressure sensors can accurately reflect static and dynamic load distributions, which can enhance their utility in healthcare settings (Wang et al., 2023).Although electrospinning sensors show great promise for clinical applications, challenges such as their long-term stability, scalability, and integration with existing medical technologies remain significant obstacles that need to be overcome before they can be widely adopted.

Currently, electrostatic spinning technology has developed a variety of sensor products for clinical applications, which are highly valuable in the field of medical monitoring and diagnosis. The following are some electrostatic spinning sensor products and their applications that have gained acceptance:Piezoelectric BiosensorPiezoelectric biosensors are commonly used to monitor physiological signals such as heart rate, pulse, and limb movement. These sensors reflect the health of the body by detecting changes in electrical resistance. For example, researchers such as Li developed a highly sensitive piezoelectric sensor for real-time heart rate monitoring (Ji et al., 2022). The sensor, made of composite nanofibers, can withstand temperatures as high as 341°C and has a maximum open-circuit voltage and short-circuit current of 184.6 V and 10.8 µA, respectively. Experimental results show that the number of pulse beats monitored is consistent with the heart rate interval of a normal person.Flexible Strain Sensor Shen and other researchers have developed a flexible strain sensor based on electrostatically spun fiber membranes with a unique three-dimensional network structure and high specific surface area. This sensor has a response range of up to 0%–630%, very high sensitivity (maximum response factor GF = 8.9 × 10^4^), and good breathability and wearing comfort (Wang J. et al., 2025). In addition, such sensors can be used in human motion monitoring and human-computer interaction systems, such as controlling the movements of robotic arms.Wearable Health Monitoring Sensors (WHS)Electrostatically spun fibers are widely used in the development of wearable sensors for continuous health monitoring. For example, researchers have developed a wearable sensor based on PLA/graphene composite nanofibers for monitoring human movement and physiological signals. This sensor has high sensitivity and fast response capability to monitor heart rate and pulse in real time (Ji et al., 2022).These electrostatic spinning sensor products not only perform well in laboratory research but are also gradually entering the clinical application stage, with some products already certified as relevant medical devices. For example, certain non-invasive wearable sensors for monitoring arterial pulse waveforms and heart rate have already passed clinical evaluation (Ji et al., 2022). The development of these technologies provides new solutions for medical monitoring and diagnosis and has broad application prospects.

## 6 Conclusions and outlooks

This paper reviews the application of electrospinning technology in the field of healthcare sensors, focusing on pressure sensors, wearable sensors, and glucose sensors. As electrospinning technology develops, more and more products prepared by this method are gradually being commercialized. The technology holds great potential in the healthcare sensor field and can be applied in every aspect of people’s lives, but it still faces some challenges. These challenges include complex sensor material manufacturing processes, high manufacturing costs, poor mechanical properties of sensor materials that cannot ensure long-term stability, and limitations that prevent large-scale development. Additionally, sensitivity and response time need further optimization. However, as time progresses, electrospinning technology continues to advance. With the discovery and use of increasingly complex materials, its application in biosensors is also improving. The future development of electrospinning technology in the sensor field can focus on solving mass production and cost issues. This will require multidisciplinary collaboration, such as in machinery, computer science, and biomedical engineering. The intersection of these disciplines with electrospinning technology will make breakthroughs easier to achieve.

Electrospun nanofiber technology holds promising applications in the field of healthcare sensors, but it still faces many challenges. From a technical standpoint, although electrospun nanofibers have demonstrated numerous excellent properties, their mechanical, electrical, and chemical stability needs further enhancement for practical applications. For example, nanofibers used in wearable sensors must be flexible and wear-resistant while maintaining high sensitivity. Additionally, the electrospinning process involves multiple parameters (e.g., voltage, flow rate, receiving distance, etc.), and precise control of these parameters is critical to ensure product quality. In the future, more advanced automated control systems need to be developed to achieve consistent fiber properties in mass production. Meanwhile, high-performance electrospun materials, such as nanofibers with biocompatibility, antimicrobial properties, and self-healing features, need to be developed for medical implants and wound dressings to meet the demands of different applications.From the perspective of industrial scale-up, the current high cost of electrospinning equipment and processes limits its large-scale application. Future technological innovations and process improvements are needed to reduce production costs, such as developing more efficient electrospinning equipment and simplifying the production process. Improving production efficiency is key to achieving industrial scale. The current production speed of electrospinning technology is relatively slow, and it is necessary to develop new technological methods, such as multi-needle electrospinning and continuous electrospinning, to enhance production efficiency. In large-scale production, ensuring the consistency and stability of product quality is crucial. Strict quality control systems and standardized production processes must be established to ensure that each fiber meets the design requirements.

The high cost of electrospinning equipment and processes currently limits its large-scale application. Future technological innovations and process improvements are needed to reduce production costs, such as developing more efficient electrospinning equipment and simplifying the production process. Improving production efficiency is key to achieving industrial scale-up. The current electrospinning technology has a relatively slow production speed, and new technical methods, such as multi-needle electrospinning and continuous electrospinning, need to be developed to enhance production efficiency. In large-scale production, ensuring the consistency and stability of product quality is crucial. Strict quality control systems and standardized production processes must be established to ensure that each fiber meets the design requirements.Although electrospun nanofiber technology has a wide range of application prospects, current market awareness and acceptance are still relatively low. Market research and product promotion are necessary to increase market awareness and demand for electrospun nanofiber products. Healthcare products require strict regulatory certification, such as FDA or CE certification. Electrospun nanofiber products must meet relevant regulatory requirements before entering the market, which demands significant investment of time and resources. Intellectual property protection is critical during commercialization. Strengthening intellectual property protection is necessary to ensure the innovation and uniqueness of the technology and to prevent infringement.In summary, electrospun nanofiber technology has broad application prospects in the field of healthcare sensors but still faces many challenges. At the technical level, further optimization of fiber properties, improvement of production process controllability, and development of new materials are needed. From the perspective of industrial scale-up, reducing production costs, improving production efficiency, and establishing strict quality control systems are essential. Regarding the commercialization path, matching market demand, passing regulatory certification, strengthening intellectual property protection, and establishing cooperative relationships with upstream and downstream enterprises are necessary. Through multifaceted efforts, the widespread application and commercialization of electrospun nanofiber technology in the field of healthcare sensors are expected to be promoted.
